# Development of national biobank for lysosomal storage disorders in India- a step towards advancing research and precision medicine

**DOI:** 10.1186/s13023-026-04195-8

**Published:** 2026-01-27

**Authors:** Jayesh Sheth, Aadhira Nair, Riddhi Bhavsar, Mahesh Kamate, Vykuntaraju K. Gowda, Ashish Bavdekar, Sandeep Kadam, Sheela Nampoothiri, Chaitanya Datar, Inusha Panigrahi, Anupriya Kaur, Siddharth Shah, Sanjeev Mehta, Sujatha Jagadeesan, Indrani Suresh, C. Ratna Prabha, Seema Kapoor, Shruti Bajaj, Radha Rama Devi, Ashka Prajapati, Koumudi Godbole, Harsh Patel, Zulfiqar Luhar, Raju C. Shah, Anand Iyer, Sunita Bijarnia-Mahay, Ratna Puri, Mamta Muranjan, Ami Shah, Suvarna Magar, Neerja Gupta, Naresh Tayade, Madhulika Kabra, Anil Jalan, Dhaval Solanki, Ashwin Dalal, Frenny Sheth, Harsh Sheth

**Affiliations:** 1 Foundation for Research in Genetics and Endocrinology, Institute of Human Genetics, Ahmedabad, India; 2 KLES Prabhakar Kore Hospital, Belgaum, India; 3 Department of Pediatric Neurology, Indira Gandhi Institute of Child Health, Bangalore, India; 4 Department of Pediatrics, K.E.M Hospital, Pune, India; 5 Department of Paediatrics, Amrita School of Medicine, Kochi, India; 6 Bharati Hospital and Research Centre, Dhankawadi, Pune India; 7 Postgraduate Institute of Medical Education and Research, PGIMER, Chandigarh, India; 8 RICN Hospital, Ahmedabad, India; 9 Department of Clinical Genetics & Genetic Counselling, Mediscan Systems, Chennai, India; 10 Department of Biochemistry, Faculty of Science, M. S. University of Baroda Vadodara, Vadodara, India; 11 Division of Genetics and Metabolism, Department of Pediatrics, Maulana Azad Medical College, Delhi, India; 12 The Purple Gene Clinic, Simplex Khushaangan, SV Road, Malad West, Mumbai, India; 13 Rainbow Children’s Hospital, Hyderabad, India; 14 Genetic Care Clinic, Ahmedabad, India; 15 Deenanath Mangeshkar Hospital & Research Centre, Pune, India; 16 Zydus Hospital & Healthcare Research Pvt Ltd, Ahmedabad, India; 17 Civil Hospital, Asarwa, Ahmedabad India; 18 Ankur neonatal hospital, Ashram Road, Ahmedabad, India; 19 Neuro Kids Clinics, Ahmedabad, India; 20 Institute of Medical Genetics and Genomics, Sir Ganga Ram Hospital, New Delhi, India; 21 Department of Pediatrics, KEM Hospital, Parel, Mumbai India; 22 BJ Wadia Hospital for Children, Parel, Mumbai India; 23 MGM Medical College, Aurangabad, India; 24 Division of Genetics, Department of Pediatrics, All India Institute of Medical Sciences, New Delhi, India; 25 Department of Paediatrics, Dr. Panjabrao Deshmukh Memorial Medical College, Amravati, India; 26 NIRMAN, Vashi, India; 27 Nirmal Mantra Children’s Hospital, Bhavnagar, India; 28 Diagnostics Division, Centre for DNA Fingerprinting and Diagnostics, Hyderabad, India

**Keywords:** Lysosomal storage disorders, Biobank, Biological specimens, Prevalence, Genotype-phenotype correlation, India

## Abstract

**Background:**

Lysosomal storage disorders (LSDs) are a diverse group of over 70 rare, inherited metabolic conditions that present significant diagnostic and therapeutic challenges, especially in genetically diverse and resource-limited settings like India. To address the lack of a centralized clinical and genomic data registry for LSDs, we established the first government-supported national LSDs biobank in India. This study describes the infrastructure, sample collection, storage procedures, ethical framework, and expected impact of the biobank on research, diagnostics, and patient care.

**Methods:**

The study includes biological samples and clinical-genetic data from 530 patients, (526 unrelated individuals and 2 sibling pairs), over a 17-year period (2008–2025). Biological samples including genomic DNA from blood, plasma, and urine precipitate were processed for enzyme and genetic investigations. A centralized webpage has been established to manage the biological sample data including clinical, enzyme and genetic data.

**Results:**

The LSD biobank cohort encompasses 8 LSD subgroups across 27 disorders, with the most common being Gaucher disease (*n* = 70), Tay-Sachs disease (*n* = 62), Mucolipidosis (ML) II/III (*n* = 44), and Morquio-A (*n* = 40). Samples originated from 15 Indian states, with a predominance of pediatric cases. Detailed phenotypic, enzymatic, and genomic profiles were generated. Enzyme assays confirmed markedly reduced activity in most cases, with variable residual activity noted in few LSDs. Genetic analyses using Sanger sequencing, PCR-RFLP, targeted gene panel sequencing, and/ or whole exome sequencing detected causative variants. Notably, c.1469T > C in the *IDUA* gene (29.4% in Hurler disease), c.230 C > G in the *GALNS* gene (22.5% in Morquio-A disease), c.1448T > C in the *GBA1* gene (56% in Gaucher disease), and c.1385 C > T and c.964G > T in the *HEXA* gene (11.3% and 8.1% respectively in Tay-Sachs disease) were the most common variants. Several novel, private mutations were also identified, broadening the mutational landscape of LSDs.

**Conclusion:**

The present study represents a scalable model for rare disease research in low- and middle-income countries. This resource lays the foundation for genotype–phenotype correlation studies, natural history analyses, and future precision medicine strategies tailored to the Indian population.

**Supplementary Information:**

The online version contains supplementary material available at 10.1186/s13023-026-04195-8.

## Background

Lysosomal storage disorders (LSDs) comprise more than 70 monogenic conditions characterized by lysosomal dysfunction [1]. It is estimated that the combined incidence of LSDs is ~ 1 in 4000 to 1 in 9000 live births as per the 2022 report by the American College of Medical Genetics and Genomics (ACMG) [2]. LSDs affect multiple organ systems, with the involvement of nervous system observed in ~ 70% of the cases [3]. They display wide spectrum of clinical manifestations with variable severity and are associated with significant morbidity and mortality.

The burden of LSDs in India is substantial, yet largely underappreciated due to the lack of widespread awareness, insufficient diagnostic infrastructure, and high costs associated with laboratory testing. In addition, delayed referrals, limited access to specialized metabolic centers, and lack of trained personnel contribute to prolonged diagnostic odysseys for many affected families. Socioeconomic barriers, such as high direct healthcare costs and inadequate insurance coverage for rare diseases, hinder access to confirmatory diagnostics and long-term care [4]. These challenges collectively contribute to underdiagnoses and delayed recognition of LSDs in the Indian setting. Sheth et al. have previously described epidemiological distribution of LSDs in India and found Gaucher disease to be the most common LSD, followed by mucopolysaccharidosis (MPS) types I and II amongst others [5–7]. The genetic diversity of the Indian population presents unique challenges and opportunities in understanding the molecular epidemiology of LSDs. While some founder mutations have been identified in specific ethnic groups such as Tay-Sachs and Morquio A disease in ethnic communities of Gujarat [8, 9], there remains a significant gap in the genetic and biochemical profiling of these disorders in the broader population context. Additionally, high cost and limited availability of enzyme replacement therapy (ERT) and substrate reduction therapy (SRT) [4, 10] further exacerbate the clinical management challenges, underscoring the need for early diagnosis, newborn screening programs, and personalized treatment approaches tailored to the Indian population.

Genetic biobanks (GBs) have long been a powerful tool in basic, translational, and clinical research, and in care practice of rare diseases [11]. The term “biobank” is defined as a structured collection of biological samples and associated data, stored for the purpose of present and future research [12]. They link a patient’s biological sample to their clinical data, providing detailed phenotypic and genotypic information. The aim is to make clinical samples and data available to the scientific community for further studies. A DNA biobank provides a centralized repository of high-quality genomic material that enables researchers to study genetic mutations, identify novel pathogenic variants, and understand genotype-phenotype correlations. Additionally, a well phenotyped population and specimens in a well-structured registry could aid in the development of diagnostic, predictive, and prognostic biomarkers as well as new treatment targets [13, 14].

The United Kingdom (UK) biobank is one of the world’s largest, with over 500,000 participants aged between 40 and 69 years [15]. Over the years, data from the UK biobank has helped global research community in unraveling the complexities in several genetic disorders like Parkinson’s disease, schizophrenia, metabolic, and neurological disorders [16–19]. Likewise, for LSDs, in addition to general biobanks, there are few independent registries and biobanks that collect genetic, clinical, and biomarker data from individuals with LSDs to help researchers understand these rare diseases better and develop potential treatments. One particular example is the Lysosomal Disease Network (LDN), a global initiative, which is a part of the Rare Diseases Clinical Research Network (RDCRN), funded by the National Institutes of Health (NIH) and led by the National Center for Advancing Translational Sciences (NCATS) [20]. Likewise, there are dedicated registries or biobanks focusing on a particular LSD. For example, the Gaucher Registry (https://www.gaucherdisease.org/blog/medical-history-international-gaucher-registry/) and the Pompe Registry (https://worldpompe.org/pompe-disease/pompe-registry/) collect data and biological samples from patients with these specific disorders [21, 22]. These registries have helped improve understanding of disease progression and response to therapies.

Recent advances in molecular biology techniques, including sequencing, and an increasing demand for well-annotated and properly preserved specimens, has led to a considerable rise in the awareness of the importance of biobank. Despite these advances, India currently lacks a structured, large-scale DNA biobank dedicated to LSDs. The absence of such a resource limits researchers’ ability to study the genetic diversity of LSD patients in India and hinders the development of precision medicine approaches tailored for the Indian population. The establishment of the LSDs biobank in India will serve as a comprehensive repository that will facilitate research into the different aspects of LSDs while integrating demographic information, enzyme activity, genetic variants and clinical data. By systematically collecting and maintaining genetic information from patients across diverse communities in the country, the biobank will support studies on disease prevalence, mutation patterns, and community specific allele frequency information and genotype-phenotype relationships. Additionally, access to demographic and enzyme data alongside DNA samples will enhance the ability to correlate genetic findings with clinical outcomes, thereby improving diagnostic accuracy and therapeutic decision-making.

In India, national efforts to address rare diseases have been strengthened through the implementation of the National Policy for Rare Diseases and the establishment of the Indian Council of Medical Research (ICMR) Rare Disease Registry (https://rdrdb.icmr.org.in/registry/). While these initiatives focus on systematic capture of clinical and demographic data, there remains a critical need for disease-specific repositories that integrate biological samples with detailed clinical, biochemical, and genetic information. To address this gap, we established the first Department of Biotechnology (DBT); Government of India funded biobank dedicated to LSDs in India. The present study describes the biobank infrastructure, sample collection and storage, ethical compliance, and its anticipated impact on research, diagnostics, and patient care. By creating a sustainable model for LSD-focused genomic biobanking, this initiative aims to accelerate therapy development focused research, expand genetic screening capabilities, serve as a resource for establishing national quality control programs, and ultimately enhance the quality of life for individuals affected by LSDs across the country.

## Materials and methods

### Study design and setup

The development of biobank for LSDs has been established as part of a comprehensive initiative aimed at advancing research into the genetic, clinical, and therapeutic aspects of LSDs. A standardized approach was implemented for the recruitment of participants, sample collection, and data management as follows. Over a period of 17 years (2008–2025), 530 patient samples were collected and included as part of the LSDs biobank initiative. This comprised of 206 female participants and 324 male participants that are diagnosed with LSD.

### Study population and participant selection

Patients were recruited from different centers and hospitals across India. The LSD subtypes included in the study were as follows: mucopolysaccharidoses (MPS), sphingolipidoses, glycogen storage disease, neuronal ceroid lipofuscinoses (NCL), glycoproteinoses, integral membrane protein disorders, lipid storage diseases, and post-translational modification defects. Inclusion criteria included individuals with a confirmed diagnosis of an LSD, based on clinical, genetics, and/or enzyme study details. An informed consent from patients or their legal guardians was obtained prior to their enrollment. Exclusion criteria included individuals who did not provide informed consent for participation.

### Collection of biological samples and storage

Biological samples including blood, plasma, and urine samples were collected for the purpose of biochemical and/or genetic tests. Blood samples were collected in EDTA tubes for DNA extraction, while plasma was separated from peripheral blood and collected in sterile containers. Urine samples were also collected from patients suspected with MPS disorders in sterile containers and were processed for quantitative and qualitative analyses of glycosaminoglycans (GAG) using the protocol as previously described [23]. High-molecular weight genomic DNA was isolated from peripheral blood sample using the salting-out method [24] and subjected for genetic studies. Genomic DNA samples, plasma samples, and urine GAG precipitates were stored at -20°C. For the purpose of biochemical enzyme assay studies, leukocytes were separated from whole blood as described previously [25] and stored at -20°C until further testing. Post-testing, all DNA and plasma samples were stored under controlled conditions to ensure long-term stability.

### Clinical, enzyme study and genetic study

Comprehensive clinical data, including age, gender, geographic region, family history, medical history, and clinical manifestations, that was documented by the referring clinician at the time of referral using a standardized clinical record form has been incorporated into the biobank. Phenotypic data were systematically coded using standardized human phenotype ontology (HPO) terminologies [26].

### Screening of LSDs and enzyme study

**Fig. 1:**
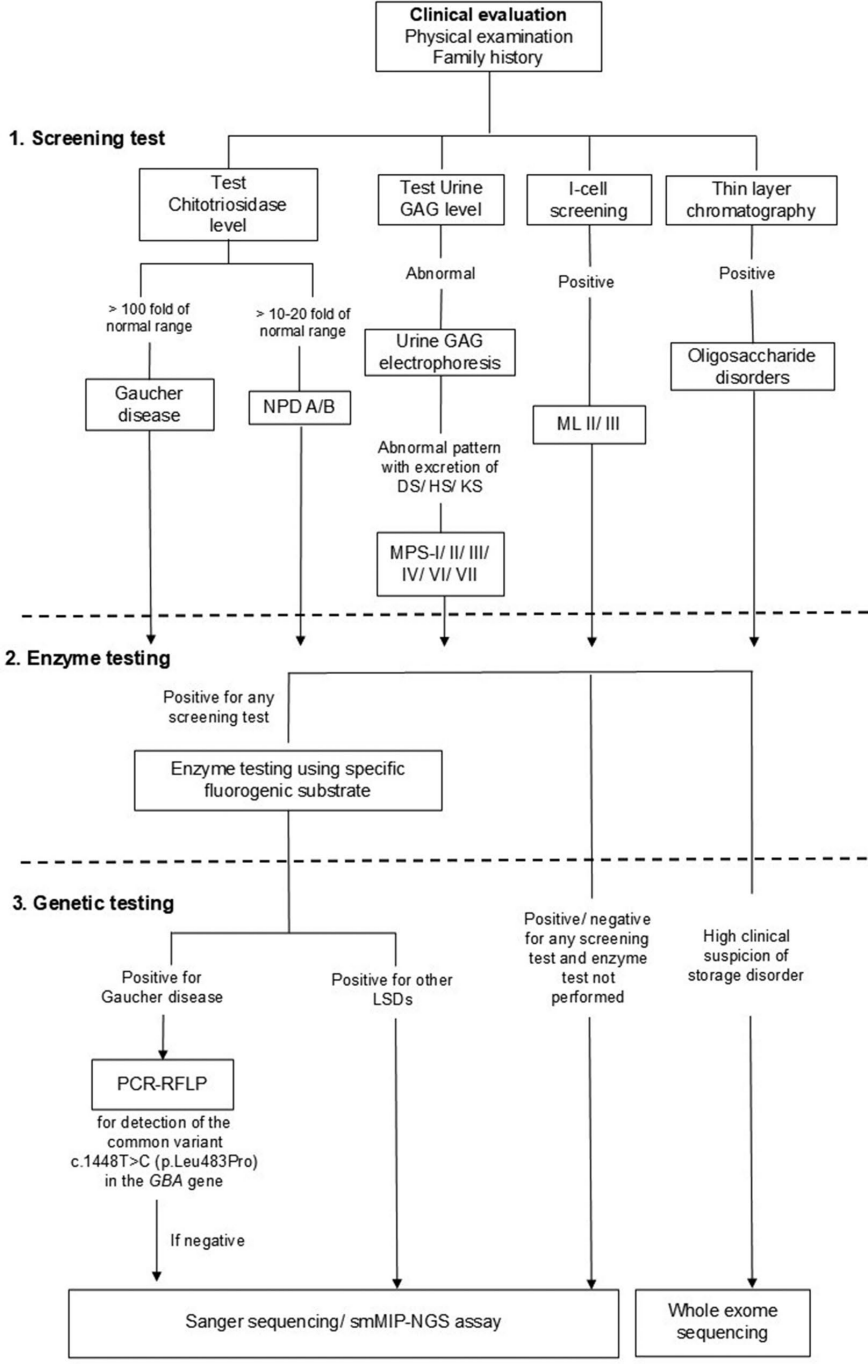
Overview of the diagnostic test pathway used for testing lysosomal storage disorders in the present cohort. GAG = glycosaminoglycan, NPD A/B = Niemann-Pick disease A/B, MPS = mucopolysaccharidosis, ML II/III = Mucolipidosis II/III, PCR-RFLP = polymerase chain reaction- restriction fragment length polymorphism, smMIP-NGS = single molecule molecular inversion probe- next generation sequencing

Primary screening of LSDs was carried out by plasma chitotriosidase, I-cell screening, and urine GAG study as per the methods described earlier [23, 27, 28]. This helped to narrow down the confirmative enzyme study from leucocytes or plasma. Enzyme assays were performed using leukocytes and/ or plasma of patients by standard protocol for a given enzyme using 4-MU fluorometric assay or p-nitrocatechol sulfate (p-NCS) spectrophotometric synthetic substrate as outlined previously [29, 30]. Following this, enzyme activity values were noted for patients diagnosed with a particular LSD. Figure 1 describes the overview of the diagnostic test pathway used for testing LSDs in the present cohort.

### Genetic screening

Genetic data were obtained using one of the testing methodologies: Sanger sequencing, targeted single molecule molecular inversion probe-next generation sequencing (smMIP-NGS) assay as developed and validated by our group, which targets 23 genes associated with 29 LSDs [31], polymerase chain reaction-restriction fragment length polymorphism (PCR-RFLP), or whole exome sequencing (WES).

### Sanger sequencing

The region of interest was amplified using specific primers designed with Primer3 tool (https://bioinfo.ut.ee/primer3-0.4.0/) [32]. Following this, bi-directional Sanger sequencing using ABI SeqStudio platform (Thermo Fisher Scientific, USA) was performed.

### Targeted single molecule molecular inversion probe (smMIP)-NGS assay

This assay was performed for 302 samples that were enzymatically diagnosed with a particular LSD. 100ng genomic DNA was used as an input for the targeted capture of genomic regions of interest of 23 genes associated with LSDs, as previously described [31]. The captured PCR products were pooled accordingly to obtain equimolar representation of all samples and were cleaned up for Illumina sequencing using XP Ampure beads. Samples were sequenced on the Illumina MiSeq platform (Illumina, USA) using custom sequencing primers and 2 × 156 bp paired-end reads.

### PCR-RFLP

In patients biochemically diagnosed with Gaucher disease, preliminary detection of the common variant c.1448T > C (p.Leu483Pro) in the *GBA1* gene was performed. For this, PCR-RFLP protocol was applied as described previously [33].

### Whole exome sequencing study

WES was carried out in 6 samples with a high index of clinical suspicion of an LSD and no genetic diagnosis was achieved using the aforementioned tests. For WES, genomic DNA of the proband was subjected to selective capture and sequencing of the protein coding regions that included exons and exon-intron boundaries of genes using either Agilent SureSelect v6 enrichment kit (Agilent, USA) or Twist Human Core Exome kit (Twist Biosciences, USA). The prepared library was subjected to paired-end sequencing with a mean coverage of ~ 100x on the Illumina HiSeq 2500 or NovaSeq 6000 platform (Illumina, USA).

### NGS data analysis pipeline

FASTQ files generated from smMIP-NGS assay and WES were aligned to the GRCh37/hg19 reference genome using BWA-MEM v0.7.12. Single nucleotide variants (SNVs) and indels were called using GATK HaplotypeCaller v4.1.2 after base quality recalibration, following the GATK best practice guidelines, as described previously [31, 34]. Copy number variants (CNVs), including single-exon events in the smMIP-NGS assay, were detected using DECoN v1.0.1 [35] with coverage normalized using minimum of 17 samples per batch for analysis. Whereas, for whole exome sequencing, CNV calls were carried out using the CNVRobot v4.0.0 tool (https://github.com/AnetaMikulasova/CNVRobot).

Variants were annotated and prioritized using Exomiser v12 [36] based on HPO-coded phenotypes, integrating multiple in silico tools (SIFT, PolyPhen-2, MutationTaster, CADD, REVEL) and databases (dbSNP, gnomAD, ClinVar). Common variants (MAF > 1%) were excluded using 1000 Genomes, TopMed, and gnomAD. Only non-synonymous and canonical splice site variants with read depth > 20x were retained. Prioritization included known pathogenic variants (ClinVar) and novel variants in known genes. Novel missense variants identified were interpreted following ACMG-AMP and ClinGen guidelines [37, 38]. Population frequency, evolutionary conservation, and in-silico predictions were evaluated to assess potential deleterious impact. Functional evidence was derived from enzyme assays performed on leukocytes or fibroblasts and from disease-specific biomarker levels, where available. For cases where parental samples were available, segregation analysis was performed to delineate the inheritance mode and confirm variant phase.

### Data management and biobank infrastructure

A secure, centralized webpage (https://geneticcentre.org/lsdbiobank/) has been established to manage both biological sample data and clinical, enzyme activity details, and genetic information. Each sample was assigned a unique identification number to ensure tracking and to link the biological samples with the corresponding clinical, enzyme and genetic data. The database ensured compliance with the data protection regulations and maintained participant confidentiality throughout the study. Data were regularly updated and validated to maintain accuracy and completeness. The biobank was structured to allow access to sample and data requests for research purposes while ensuring that all materials were utilized in compliance with ethical guidelines and participant consent agreements.

### Ethical and legal considerations

The institutional ethics committee of the Foundation for Research in Genetics and Endocrinology (FRIGE) approved the study at the Institute of Human Genetics (Approval ID: FRIGE/IEC/19/2021). A written informed consent for the study was obtained from the guardians of all the participating subjects as per the 1975 Helsinki declaration. Measures were implemented to ensure that all data and biological samples were anonymized to protect participant identity. The study is compliant with local and international regulations regarding data privacy, including the General Data Protection Regulation (GDPR).

## Results

A total of 530 patient samples that were collected and documented over the period of 17 years (2008–2025) were included as part of the national biobank initiative for lysosomal storage disorders (LSDs), comprising 526 unrelated individuals and two sibling pairs. We observed consanguinity in 13.6% (*n* = 72/530) of the total cases and in 12% (*n* = 64/530), there was no reported consanguinity. However, in the remaining 394 cases, consanguinity status was unknown due to lack of clinical or ethnic origin history. The enrolled cohort spans a diverse spectrum of LSDs, with eight different LSD subgroups and 27 different LSDs represented in the registry. All LSDs included in the study design show autosomal recessive mode of inheritance, except MPS II and Fabry disease which follow X-linked recessive inheritance pattern.

**Fig. 2:**
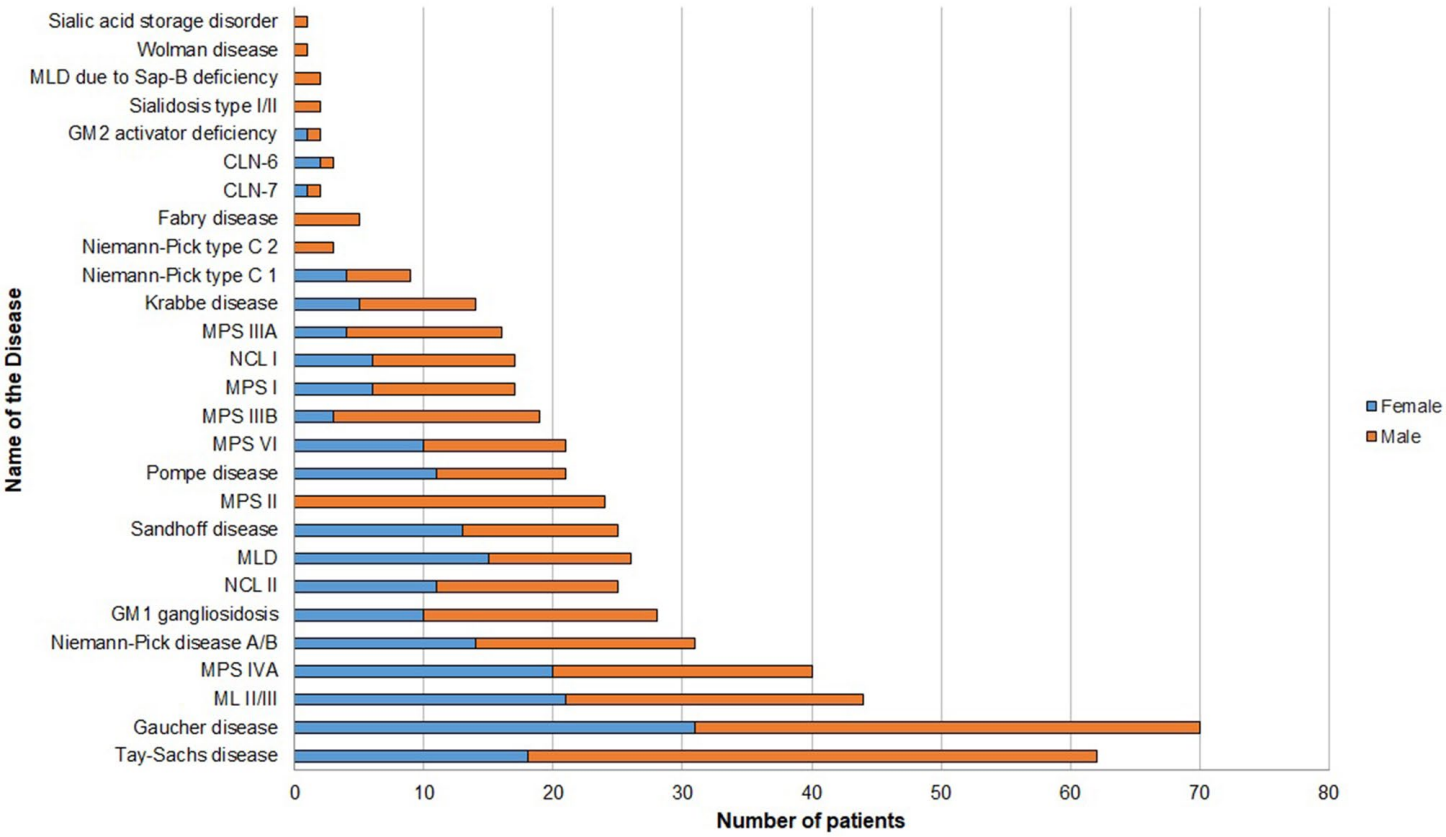
Disease-wise and sex-wise distribution of patient samples in the LSDs biobank

The highest number of patient samples in the LSDs biobank were of sphingolipidoses subgroup (*n* = 265; 50%), with maximum cases of Gaucher disease (*n* = 70; 26%), followed by Tay-Sachs disease (*n* = 62; 23%), and Mucolipidosis (ML) II/III (*n* = 44; 16.6%). The second largest disease subgroup was MPS (*n* = 137; 25.8%), of which Morquio A disease (*n* = 40; 29.2%) represented majority cases in the biobank. Of note, LSDs such as Niemann-Pick disease A and B (*n* = 31; 5.8%), GM1 gangliosidosis (*n* = 28; 5.3%), and Sandhoff disease (*n* = 24; 4.5%) contributed significantly to the biobank. Importantly, the biobank also consists of patient samples with rare LSDs namely Niemann-Pick type C1, Niemann-Pick type C2, neuronal ceroid lipofuscinosis type 6, neuronal ceroid lipofuscinosis type 7, metachromatic leukodystrophy due to saposin B deficiency and GM2 activator deficiency. Figure 2 describes the disease-wise distribution of patient samples in the LSDs biobank.

**Fig. 3:**
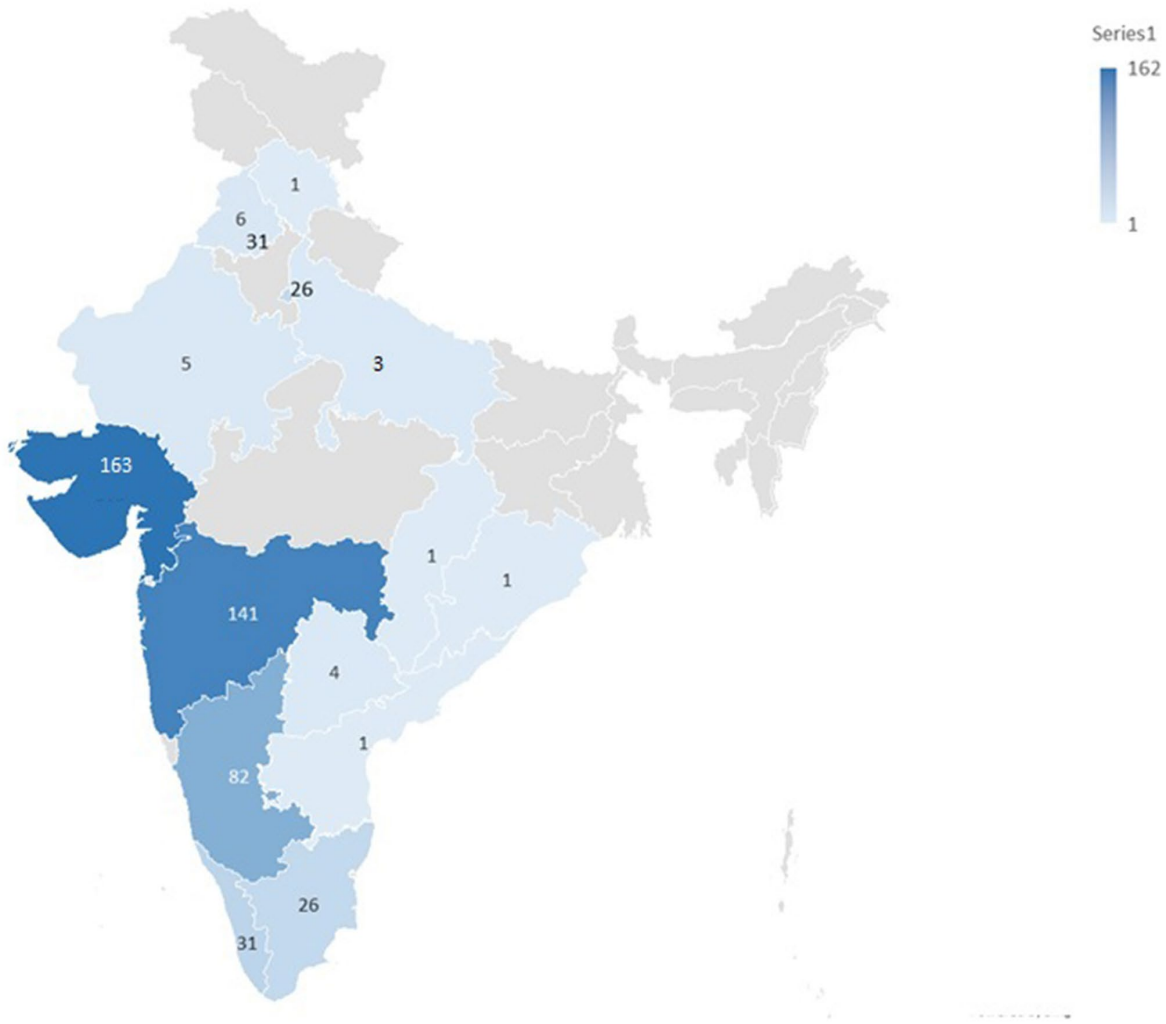
State-wise distribution of patient samples in the LSDs biobank

Patients were referred from fifteen different geographical states of India, reflecting a wide national coverage. Importantly, the majority samples represented in the LSDs biobank were from Gujarat (*n* = 162), Maharashtra (*n* = 141), and Karnataka (*n* = 82), followed by Kerala (*n* = 31), Chandigarh (*n* = 31), New Delhi (*n* = 26), and Tamil Nadu (*n* = 26). Figure 3 depicts the state-wise distribution of patient samples in the LSDs biobank. All the patient samples included in the study were either directly recruited at the FRIGE Institute of Human Genetics, Ahmedabad or were referred from other hospitals, genetic laboratories or institutes from different geographical states across India. Additional file 1 provides details of the major referring sites that contributed patient samples in the LSDs biobank.

**Fig. 4:**
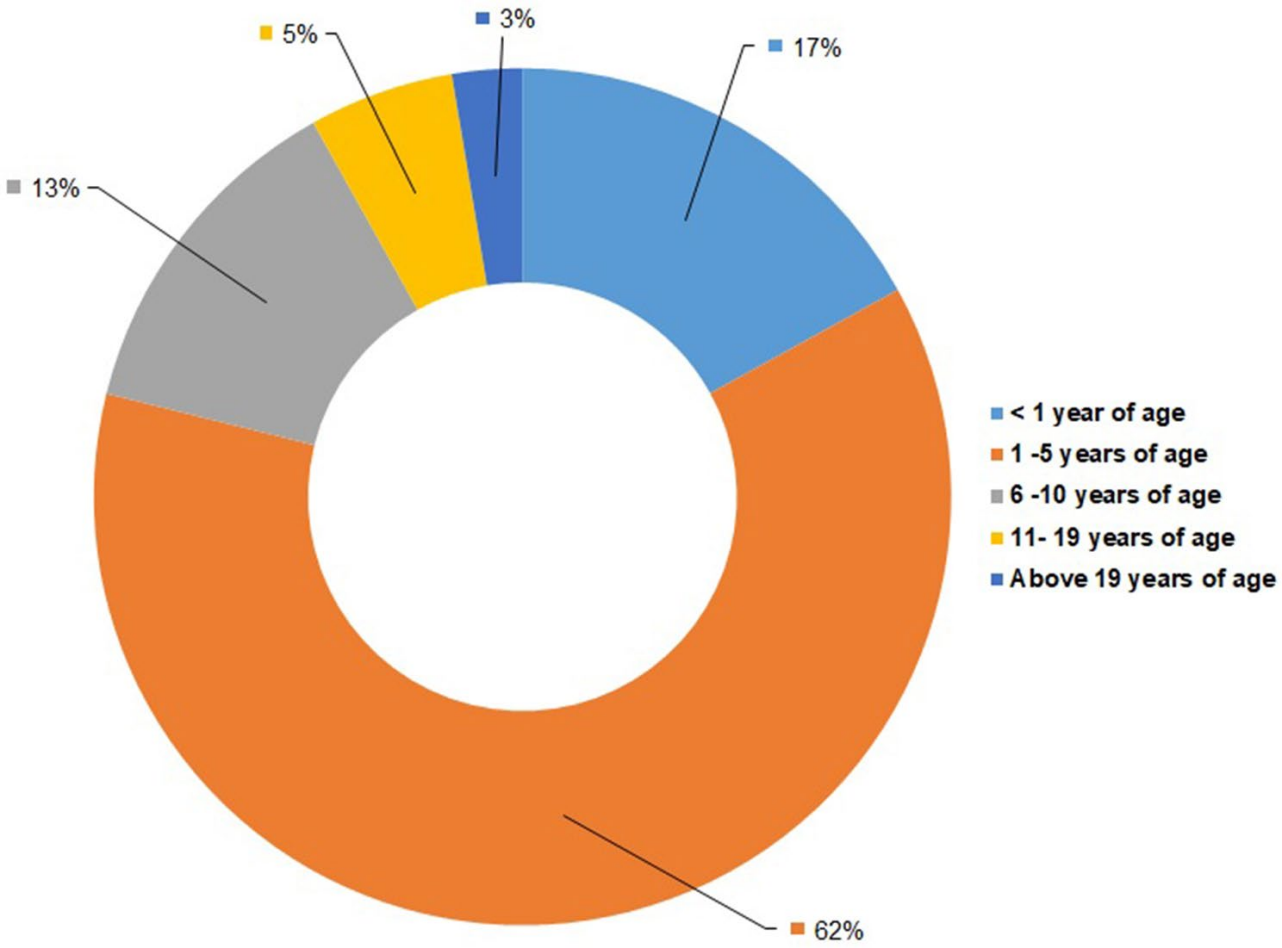
Age-group wise distribution of patient samples in the LSDs biobank

A disease-wise distribution analysis showed marked differences in the prevalence of specific LSDs across different states. A chi-square test revealed a significant association between LSD subtype and geographic region (χ² = 38.91, *p* = 0.000109) (Additional file 2). The distribution of LSD subtypes was non-uniform across regions, with NCL contributing most to the observed deviation, particularly due to an excess of cases in the southern region. Sphingolipidoses also demonstrated regional variation, with higher-than-expected representation in the western region. Other LSD categories showed comparatively minor deviations from expected distributions. These findings indicate regional heterogeneity in the distribution of LSD subtypes within the cohort. Age at diagnosis for all patients was noted and we found that 62% (*n* = 328/530) of the total patient samples in the biobank belonged to the age group of 1 to 5 years. Figure 4 gives age-group wise distribution of patient samples included in the LSDs biobank. A chi-square test revealed a significant association between LSD subtype and age at presentation (χ² = 96.15, *p* < 0.00001) (Additional file 2). The distribution of LSD subtypes was non-uniform across age groups, with sphingolipidoses showing a predominance in the < 1 year and 1–5-year age groups and a marked underrepresentation in the 6–10-year group. In contrast, mucopolysaccharidoses were significantly overrepresented in the 6–10-year age group and less frequent in infancy. NCL was more commonly observed in the 1–5-year age group, while other LSD categories showed minor age-related deviations. These findings indicate substantial age-dependent heterogeneity in the distribution of LSD subtypes within the cohort. Sex-data for all the patient samples revealed a higher percentage of males (61.1%, *n* = 324/530) as compared to females (38.9%, *n* = 206/530). The sex-wise distribution for each LSD is represented in Fig. 2.

Clinical details were analyzed for patients across various LSDs. Overall, the most common clinical signs included coarse facial features, hepatosplenomegaly, developmental regression, skeletal abnormalities and short stature. Distinct patterns were noted when stratified by disease type. For example, coarse facial features, short stature and dysostosis multiplex were common signs among patients affected with MPS disorders and in patients with ML II/III. Hepatosplenomegaly was a key observation in patients affected with Gaucher disease, Niemann-Pick disease and GM1 gangliosidosis. Developmental regression was noted in patients affected with GM1 and GM2 gangliosidosis, Krabbe disease, MLD, and neuronal ceroid lipofuscinoses (NCL I and II). Cherry red spot was commonly seen in patients with GM1 gangliosidosis, Tay-Sachs disease, and Sandhoff disease. Cardiomyopathy along with respiratory insufficiency and hypotonia were distinct features noted among patients with Pompe disease.

Lysosomal enzyme activity data are available for 88% (*n* = 468/530) cases in the LSDs biobank. Enzyme activity assays performed across different LSD types demonstrated low residual activity in patient samples as compared to that observed in normal individuals (Table 1). The percentage residual activity was estimated relative to the lower limit of the normal reference range and was observed to be generally below 25% for most of the LSD types, consistent with severe enzyme deficiency. Notably, MPS II, MPS IIIA and IIIB had near-complete loss of activity (0–0.5%), while Fabry disease, MPS VI, Gaucher disease, Niemann-Pick disease A/B exhibited relatively higher residual activity (10–20%), suggesting variability in enzyme deficiency across LSDs.

**Table 1:** Overview of enzyme assay results in affected patient samples for 17 LSDs

Sr No	Disease name	Enzyme name	Number of samples	Enzyme activity (mean *+* SD) (nmol/hr/mg protein)	Median (nmol/hr/mg protein)	Percentage residual activity in affected patient samples (%)	Normal range (nmol/hr/mg protein)
1	MPS I	α-iduronidase	14	0.42 *±* 0.37	0.4	6.56	6.1 - 23.9
2	MPS II^a^	α-iduronidase-sulfatase	21	11.8 *±* 17.8	2.6	0.65	400 - 1400
3	MPS IIIA	Heparan sulphamidase	8	0.14 *±* 0.23	0	0.00	2.1 - 9.5
4	MPS IIIB^a^	N-Acetyl-α-D-glucosaminidase	9	1 *±* 1.46	0.3	0.51	58.6 - 258
5	MPS IVA	β-galactosidase-6-sulphate-sulphatase	32	0.50 *±* 0.50	0.33	11.80	2.8 - 42.6
6	MPS VI	Arylsulfatase B	17	0.15 *±* 0.11	0.13	20.00	0.65 – 8.5
7	Gaucher	β-glucocerebrosidase	67	1.15 *±* 0.7	1.1	27.50	4.0 - 32.8
8	Niemann-Pick disease A/B	Sphingomyelinase	28	0.71 *±* 0.28	0.79	43.89	1.8 - 9.6
9	Pompe^b^	α-1,4-glucosidase	21	0.22 *±* 0.09	0.22	NA	0.29 - 0.68
10	Krabbe	β-galactocerebrosidase	13	1.02 *±* 1.6	0.68	22.67	3.0 - 29.5
11	MLD	Arylsulfatase A	24	0.11 *±* 0.08	0.09	15.00	0.6 - 4.9
12	Fabry	α-galactosidase	5	2.09 *±* 0.89	1.75	21.60	8.1 - 28.5
13	GM1 gangliosidosis	β-galactosidase	26	1.25 *±* 0.99	1	6.67	15.0 - 285
14	Tay-Sachs disease	β-hexosaminidase A	61	4.5 *±* 3.7	3.7	5.90	62.7 - 659.4
15	Sandhoff disease	β-hexosaminidase A	23	31.2 *±* 18.3	30.8	49.01	62.7 - 659.4
		β-hexosaminidase total		50.5 *±* 29.3	51.3	26.60	192.7 - 1758.7
16	NCL I	Palmitoyl-protein thioesterase 1 (PPT1)	13	1.2 *±* 1.4	0.58	7.44	7.8 - 134.1
17	NCL II	Tripeptidyl peptidase 1 (TPP1)	20	4.8 *±* 4.2	4.5	8.31	54.1 - 414.3

^a^Enzyme activity was carried out in plasma and activity was expressed in nmol/h/ml plasma

^b^Enzyme activity is expressed by calculating ratio of with acarbose and without acarbose

NA: not applicable

MPS: mucopolysaccharidosis

MLD: metachromatic leukodystrophy

NCL: neuronal ceroid lipofuscinosis

Genetic analyses were performed using either one of the four different methodologies, namely, Sanger sequencing, PCR-RFLP, targeted smMIP panel or WES. A total of 192 samples were assessed by Sanger sequencing, 302 samples by targeted smMIP-panel study, 30 samples by PCR-RFLP and 6 samples by WES. We identified multiple pathogenic and likely pathogenic variants across different LSD subtypes, reflecting the genetic heterogeneity of these disorders (Table 2). Majority of these variants detected have been previously reported in the literature. In addition to these variants, several private mutations have also been identified in the LSD genes, which have not been previously reported in the global database. Additional file 3 provides enzyme and molecular details for all the patient samples in the biobank. Notably, in four cases, enzyme assays confirmed a biochemical diagnosis of LSD; however, the smMIP-NGS assay did not identify any causative variants. These included one case each of MPS II and Niemann-Pick disease A/B and two cases of ML II/III. As all these cases were evaluated using the standard diagnostic workflows in place at the time of testing, further orthogonal molecular testing for investigating other variant types including large genomic rearrangements and variants in the deep intronic or regulatory regions of the gene were not performed.

**Table 2:** Common genetic variants identified for 16 diseases in the LSDs biobank

Sr no	Disease name (OMIM)	Gene	Codon change	Amino acid change	Zygosity	Percentage of patients in the biobank	Geography/ ethnicity	Reported/Novel variant	Classification
1	MPS I (#607014)	*IDUA*	c.1469T> C	p.Leu490Pro	Homozygous	29.4% (*n*=5/14)	Pan-India	Reported	Pathogenic
			c.1855 C> T	p.Arg619Ter	Homozygous	14.3% (*n*=2/14)	Pan-India	Reported	Pathogenic
2	MPS II (#309900)	*IDS*	c.263G> A	p.Arg88His	Hemizygous	8.7% (*n*=2/23)	Gujarat	Reported	Pathogenic
			c.1403G> A	p.Arg468Gln	Hemizygous	8.7% (*n*=2/23)	Pan-India	Reported	Pathogenic
3	MPS III A (#252900)	*SGSH*	c.1129 C> T	p.Arg377Cys	Homozygous	12.5% (*n*=2/16)	Karnataka	Reported	Pathogenic
			c.571G> A	p.Gly191Arg	Homozygous	12.5% (*n*=2/16)	Gujarat	Reported	Pathogenic
4	MPS III B (#252920)	*NAGLU*	c.1694G> T	p.Arg565Leu	Homozygous	15.8% (*n*=3/19)	Southern India	Reported	Pathogenic
			c.291T> G	p.Cys97Trp	Homozygous	10.5% (*n*=2/19)	Gujarat	Reported	Likely pathogenic
5	MPS IV A (#253000)	*GALNS*	c.230 C> G	p.Pro77Arg	Homozygous	22.5% (*n*=9/40)	Gujarat	Reported	Pathogenic
			c.647T> C	p.Phe216Ser	Homozygous	10% (*n*=4/40)	Pan-India	Reported	Pathogenic
			c.107T> G	p.Leu36Arg	Homozygous	5% (*n*=5/40)	Gujarat	Reported	Pathogenic
6	MPS VI (#253200)	*ARSB*	c.352_365dup	p.Pro123Serfs*16	Homozygous	33% (*n*=7/21)	Gujarat	Reported	Pathogenic
			c.904G> A	p.Gly302Arg	Homozygous	14.2% (*n*=3/21)	Pan-India	Reported	Pathogenic
7	Pompe (#252300)	*GAA*	c.1 A> G	p.Met?	Homozygous	14.2% (*n*=3/21)	Kerala	Reported	Pathogenic
8	Gaucher (#230800)	*GBA1*	c.1448T> C	p.Leu483Pro	Homozygous	50% (*n*=35/70)	Pan-India	Reported	Pathogenic
			c.1448T> C	p.Leu483Pro	Compound heterozygous	12.8% (*n*=9/70)	Pan-India	Reported	Pathogenic
9	Niemann-Pick disease A/B (#257200/ #607616)	*SMPD1*	c.1624 C> T	p.Arg542Ter	Homozygous	19.3% (*n*=6/31)	Pan-India	Reported	Pathogenic
10	MLD (#250100)	*ARSA*	c.1492dup	p.Leu498Profs*10	Homozygous	7.7% (*n*=2/26)	Pan-India	Reported	Pathogenic
11	GM1 gangliosidosis (#230500)	*GLB1*	c.569G> T	p.Gly190Val	Homozygous	7.1% (*n*=2/28)	Gujarat	Novel	Pathogenic
12	Tay-Sachs disease (#272800)	*HEXA*	c.1385 A> T	p.Glu462Val	Homozygous	11.3% (*n*=7/62)	Gujarat	Reported	Pathogenic
			c.1277_1278insTATC	p.Tyr427Ilefs*5	Homozygous	8.1% (*n*=5/62)	Gujarat	Reported	Pathogenic
			c.964G> T	p.Asp322Tyr	Homozygous	8.1% (*n*=5/62)	Gujarat	Reported	Pathogenic
13	Sandhoff disease (#268800)	*HEXB*	c.850 C> T	p.Arg284*	Homozygous	20% (*n*=5/25)	Pan-India	Reported	Pathogenic
			c.534_541delAGTTTATC	p.Val179Argfs*10	Homozygous	8% (*n*=2/25)	Pan-India	Reported	Likely pathogenic
14	NCL I (#256730)	*PPT1*	c.713 C> T	p.Pro238Leu	Homozygous	47% (*n*=8/17)	Karnataka	Reported	Pathogenic
			c.674T> C	p.Phe225Ser	Homozygous	11.8% (*n*=2/17)	Pan-India	Reported	Pathogenic
15	NCL II (#204500)	*TPP1*	c.616 C> T	p.Arg206Cys	Homozygous	26.9% (*n*=7/26)	Pan-India	Reported	Pathogenic
			c.617G> A	p.Arg206His	Homozygous	7.7% (*n*=2/26)	Karnataka	Reported	Pathogenic
16	Mucolipidosis II/III (#252500/ #252600)	*GNPTAB*	c.3503_3504del	p.Leu1168Glnfs*5	Homozygous	29.5% (*n*=13/44)	Pan-India	Reported	Pathogenic

MPS: mucopolysaccharidosis

MLD: metachromatic leukodystrophy

NCL: neuronal ceroid lipofuscinosis

## Discussion

The present study describes the first national-level initiative in India to develop a dedicated biobank for LSDs, a group of rare, multisystem disorders with significant diagnostic and therapeutic challenges. While international registries such as the International Collaborative Gaucher Group (ICGG) registry, the Fabry registry, the Pompe registry, and the Hunter Outcome Survey (HOS) [21, 22, 39, 40], have contributed significantly in understanding the natural history and treatment outcomes in LSDs, these registries predominantly represent data from North America, Europe and other high-income regions. In addition, the scope of rare disease registries without bio-specimen collection is inherently limited to observational research. In contrast, registries integrated with biobanks provide a substantially broader scientific utility by enabling molecular analyses, biomarker discovery, validation of diagnostic assays, identification of novel disease causing variants, and translational research. A systematic review by Garcia et al. demonstrated that biobank-linked registries combine the strengths of stand-alone registries and biobanks, resulting in greater research productivity and translational impact in rare diseases [41]. In this context, the present LSD biobank represents a significant advancement beyond data-only registries by integrating biological samples from a broad and diverse Indian population with well-curated clinical, enzymatic, and genetic data, thereby enabling precision medicine approaches and therapy-oriented research. Its strengths lie in longitudinal sample preservation, inclusion of enzyme-confirmed and genetically diagnosed cases, and representation of population-specific variant spectrum that is linked with HPO coded phenotype data. By encompassing multiple LSDs across diverse regions and socioeconomic backgrounds, the biobank provides insights into natural history, diagnostic delays, and genotype–phenotype correlations specific to the Indian population that are underrepresented in existing international datasets. This biobank represents a unique and valuable resource for studying the epidemiology of LSDs, disease characteristics, genetic diversity, and the development of novel therapeutic possibilities, particularly in underrepresented populations such as those in South Asia. In the Indian context, where access to advanced testing remains uneven, this biobank provides a scalable framework to support molecular epidemiology research, policy implementation, and precision medicine initiatives for rare diseases.

The present LSDs biobank comprises patient samples from several government hospitals as well as private clinics, across states representing large geographic and ethnic diversity of India, particularly from Western and Southern India and some from Northern India [42]. A higher representation of patients from Gujarat and Maharashtra was observed in the biobank, which may be attributed to easier access to diagnostic facilities, greater referral linkage with the center, and greater awareness of genetic testing. Notably, founder variants have been previously documented in two communities from Gujarat for Tay-Sachs disease and Morquio-A syndrome [8, 9], which may also partly account for the regional clustering of these cases, while Gaucher disease is seen pan-India. In contrast, recruitment of patients into the biobank from the eastern part of the country is lower than anticipated based on the population size of this region. Literature data also shows a lack of comprehensive data on LSDs from the eastern states of the country [7]. This is partly attributable to limited public awareness and scarcity of well-established diagnostic centers across Eastern India. Recently, Institute of Post Graduate Medical Education & Research (IPGMER) in Kolkata is designated as a Centre of Excellence (CoE) for rare diseases in Eastern India, under the National Policy for Rare Diseases, 2021. This is likely to improve the future representation of LSD patients from this region of the country. Overall, the geographic distribution of samples in the biobank reflects the current availability of diagnostic facilities, referral networks, and clinician awareness, which remain uneven across India. In addition to variability in clinical awareness and access to specialized metabolic testing, logistical constraints such as long-distance sample transport, cold-chain maintenance, and limited courier connectivity from remote regions pose significant challenges to sample collection. Furthermore, linguistic diversity and language barriers can hinder effective communication between healthcare providers, patients, and referral centers, potentially affecting consent processes, follow-up, and timely sample submission [43]. Consequently, underrepresentation from certain states is more likely attributable to these systemic and infrastructural barriers rather than true differences in disease prevalence. Addressing these challenges through regional capacity building, multilingual engagement, and decentralized sample collection models will be critical for improving nationwide representation in future phases of the biobank.

In the present cohort, the most common LSD identified was Gaucher disease under the sphingolipidoses subgroup. This observation is consistent with prior Indian studies and international cohorts, where Gaucher disease and MPS subtypes have been reported as predominant LSDs [6, 44–46]. Among the MPS subgroup, the prevalence of Morquio A (MPS IVA) in the present biobank was recorded to be highest. This is probably because of the founder variant in the *GALNS* gene in the Gujarati-Indian population [9]. Majority of the multi-center studies from India as well as European and North American cohorts of LSD patients have shown a relatively low prevalence of Tay-Sachs and GM1 gangliosidosis in comparison to more common Gaucher disease and MPS subtypes [45, 47–49]. However, our cohort differs in this regard, as there is a high proportion of cases with Tay-Sachs disease. This could be due to the presence of previously reported founder variant in the *HEXA* gene in the Gujarati community [8]. Unlike earlier reports on the prevalence of LSDs, which relied primarily on enzyme-based diagnosis of LSDs, our study has utilized enzyme testing coupled with confirmatory genetic testing. This allowed for improved diagnosis and identification of even rare LSDs like GM2 activator protein deficiency, ceroid lipofuscinosis type 6, ceroid lipofuscinosis type 7, Niemann-Pick type C1, Niemann-Pick type C2 and sialidosis. Improved molecular diagnostics and a higher index of suspicion have contributed to identifying these rare LSD types in the country. Overall, these differences in the prevalence of LSDs in the present study highlight the need for increased awareness among clinicians about these rare LSDs and a large multi-centric dataset to understand their burden and distribution.

The mean age at diagnosis in our cohort showed considerable variability across different LSD types. This suggests there are disease-specific onset patterns and in some cases, a delay in clinical recognition. The mean age at diagnosis for patients with Gaucher (5.72 years) was younger than that reported in previous cohorts [46]. Increased awareness of Gaucher disease and improved diagnostic tests may account for this difference. Diseases such as GM1 gangliosidosis, Tay-Sachs disease and Sandhoff disease predominantly presented in infancy and early childhood, with their rapid neurodegenerative course and early onset of symptoms. This is in concordance with the observation in earlier cohorts [50, 51]. Fabry disease and MPS types IIIA/B, demonstrated a relatively higher frequency in older children and adolescents, which may reflect their more attenuated or slowly progressive forms and overlapping clinical features that lead to delayed recognition. These findings emphasize the importance of maintaining a high index of suspicion for LSDs across different age groups, as well as the need for awareness and screening strategies to facilitate earlier diagnosis and intervention.

Adult patients with LSDs are underrepresented in the present biobank cohort. This observation is consistent with the existing literature, which reports a predominance of cases in the pediatric age group [52]. In the current dataset, 79% (*n* = 417/530) of patients were below 5 years of age at the time of diagnosis. This skew toward the pediatric population is likely attributable to the composition of participating centers, the majority of which are pediatric institutions, with limited representation from adult care facilities. Furthermore, due to the underlying disease biology of many LSDs, clinical manifestations tend to be more severe and rapidly progressive in pediatric cases, leading to earlier recognition and referral. In contrast, adult-onset or attenuated forms are often associated with partial residual enzyme function, hypomorphic variants, or modifying genetic and environmental factors, leading to slower substrate accumulation and milder, organ-restricted manifestations [53]. Such patients may present with non-specific or isolated symptoms including skeletal involvement, neuropathy, cardiomyopathy, or hepatosplenomegaly rather than the classical multisystem phenotype seen in childhood. As a result, these patients are frequently misdiagnosed or remain undiagnosed. For example, late-onset Pompe disease or Fabry disease may be misdiagnosed as neuromuscular disorders or dermatological disorders, respectively [54, 55]. Also, there is a growing recognition of neuropsychiatric features in adult LSDs, as Nijmeijer et al. [56] has recently reported behavioral and psychiatric symptoms such as attention deficit hyperactivity disorder (ADHD) and aggressiveness common in adult patients with MPS III. These observations underscore the importance of awareness among psychiatrists and neurologists regarding the heterogeneous clinical presentation of adult LSDs. Thus, this diagnostic gap likely contributes to the underrepresentation of adult cases in the biobank and should be considered when interpreting national disease patterns, natural history, and prevalence estimates. Expanding biobank participation to include adult clinics and fostering collaboration with neurology specialists will be essential to improve the ascertainment of adult LSD cases.

Enzyme activity was quantified in patient leukocyte samples using a fluorometric assay, with reference ranges established from internally validated control data. The residual enzyme activity levels observed in affected individuals within our cohort were consistent with values reported in previous studies. For example, near-absent α-iduronate sulfatase activity in individuals with MPS II and markedly reduced β-glucosidase activity (typically < 5% of normal) in Gaucher disease have been documented in earlier patient cohorts [57]. Variability in residual enzyme activity across different LSDs may reflect differences in age at diagnosis, underlying genotype, or disease severity. These findings highlight the need for integrated analyses involving molecular data and long-term clinical follow-up. Notably, one patient with Niemann-Pick disease type C in the biobank demonstrated reduced β-glucosidase activity. This finding is consistent with prior reports indicating that false low β-glucosidase activity can occur in Niemann-Pick type C, potentially due to secondary biochemical effects unrelated to primary enzyme deficiency [58].

Genetic analyses identified pathogenic/ likely pathogenic variants in the LSD genes, with a predominance of missense mutations and a high rate of homozygosity. Recurrent variants such as c.1469T > C (p.Leu490Pro) in the *IDUA* gene, and c.1448T > C (p.Leu483Pro or p.L444P) in the *GBA1* gene, have been previously reported in Indian cohorts [59, 60] and are consistent with known mutational hotspots or founder effects. The presence of such recurrent variants underscores the influence of ancestry, endogamy, and regional population structure on the distribution of LSD-associated mutations in India [42]. These findings have important diagnostic implications, as incorporation of population-relevant variants into targeted molecular testing strategies may facilitate rapid confirmation following biochemical diagnosis, particularly in resource-limited settings. In addition, recognition of recurrent variants supports cascade testing and carrier screening within affected families and communities. At a broader level, population-specific variant frequency data contribute to risk stratification and may inform the design of future newborn screening or early diagnostic programs in the country. The detection of novel or rare variants further underscores the potential of the biobank to contribute to global variant databases such as ClinVar, especially for underrepresented populations. Recently, a research group in the UK expressed interest in a rare case of ceroid lipofuscinosis type 7, from the LSDs biobank that was uploaded to the ClinVar database. This case was subsequently integrated into a larger dataset comprising similar cases from multiple centers to advance collective understanding of the condition [61]. This illustrates the utility of the biobank in facilitating genotype–phenotype correlation studies. Moreover, samples from this biobank were instrumental in the development and validation of a novel diagnostic protocol utilizing smMIP-NGS technology [31].

Importantly, in four enzymatically diagnosed LSD cases, no causative variants were identified using the smMIP-NGS assay. The lack of molecular confirmation in these biochemically positive cases may be attributable to unassessed variant classes, including large genomic rearrangements, deep intronic or regulatory variants, or other complex genetic mechanisms not captured by the applied methods. Retention of these cases within the biobank preserves their value for future comprehensive molecular re-evaluation as technologies and analytical approaches continue to advance [62].

The resources collated and developed through this national LSD biobank are intended to support collaborative research by the wider scientific and clinical community, while ensuring ethical oversight and responsible use of biological materials and associated data. Investigators seeking access to bio-specimens or curated datasets are required to submit a structured proposal outlining the scientific objectives and requirement for specific resources. A detailed description of the application process and link to the online submission form can be found at https://geneticcentre.org/lsdbiobank/#data-access. Proposals are reviewed by the LSD Biobank Steering Committee, which evaluates scientific merit, relevance to rare disease research, ethical compliance, and potential impact on sample availability. On approval, materials data transfer agreement is signed. Approved projects are supported through coordinated data and sample management mechanisms, facilitating efficient utilization of resources while maintaining participant confidentiality and regulatory compliance. This structured access model ensures that the biobank functions as a sustainable, high-quality national resource beyond the initial funding period. Supported studies should include LSD biobank authors as appropriate, based on their qualifying contributions in their report. A collaborative effort has been initiated with the Tata Institute for Genetics and Society (TIGS), Bengaluru, which involves the use of biobank-derived samples to develop human stem cell–based disease models for rare genetic disorders. These stem cell-derived models are expected to facilitate the understanding of disease mechanisms and to serve as platforms for the development and evaluation of potential therapeutic interventions. In addition, the Centre for DNA Fingerprinting and Diagnostics (CDFD), Hyderabad is utilizing biobank-derived samples for the development of mass spectrometry-based assays to assess specific LSDs in patient samples.

With standardized procedures for sample collection, DNA preservation, and data capture, this biobank represents a scalable model for other rare disease biorepositories in low- and middle-income countries. The integration of clinical, enzymatic, and genomic data within this biobank framework enables its application in natural history studies, clinical trial readiness, and precision medicine initiatives for LSDs. These efforts are aligned with the Rare Disease Policy of the Government of India [63]. However, this is an early-phase report, and there are several limitations. Sample size for certain LSD subtypes remains limited, and long-term follow-up data is pending. Standardization of clinical phenotyping across referring centers remains a challenge. We aim to expand the reach to include additional regions and institutions, integration with national rare disease registries, inclusion of longitudinal clinical data and patient-reported outcomes, development of access and governance frameworks aligned with standard guidelines and inclusion of the adult population.

## Conclusion

The national LSDs biobank represents a critical milestone for rare disease infrastructure in India. It enables multidimensional insights into the clinical and genetic architecture of LSDs and provides a foundational platform for collaborative research. This rich resource will be available to the international community of scientists interested in studying LSDs for many years to come. Its continued development and integration into broader rare disease ecosystems hold promise for improving diagnosis, care, and novel therapy development in India and beyond.

## Supplementary Information

Below is the link to the electronic supplementary material.

Supplementary Material 1

Supplementary Material 2

Supplementary Material 3

## Data Availability

All data supporting the findings of this study are available within the paper and it’s supplementary information.

## Abbreviations

LSD: Lysosomal storage disorder
MPS: Mucopolysaccharidosis
GAG: Glycosaminoglycan
smMIP: Single molecule molecular inversion probe
NGS: Next generation sequencing
PCR-RFLP: Polymerase chain reaction-restriction fragment length polymorphism
WES: Whole exome sequencing
NCL: Neuronal ceroid lipofuscinosis
HPO: Human phenotype ontology
SNV: Single nucleotide variant
CNV: Copy number variant
