# Supplementary figures and images for: Development of national biobank for lysosomal storage disorders in India- a step towards advancing research and precision medicine
Source: Orphanet J Rare Dis. 2026 Jan 27;21:65. doi: 10.1186/s13023-026-04195-8 (PMC12918591; doi:10.1186/s13023-026-04195-8)

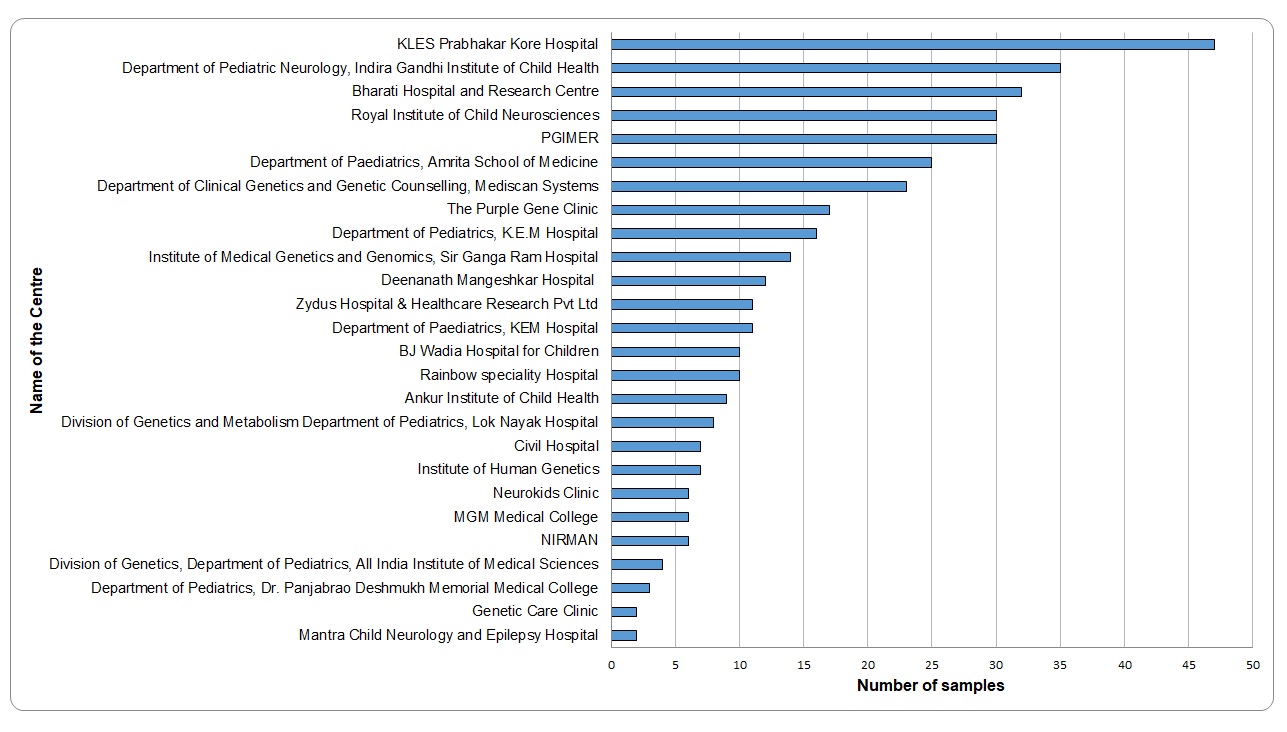

Supplement: Supplementary file 1 — Supplementary Material 1 [file 13023_2026_4195_MOESM1_ESM.jpg]
